# Sulfur speciation by HPLC-ICPQQQMS in complex human biological samples: taurine and sulfate in human serum and urine

**DOI:** 10.1007/s00216-018-1251-z

**Published:** 2018-07-30

**Authors:** Bassam Lajin, Walter Goessler

**Affiliations:** Institute of Chemistry – Analytical Chemistry for Health and Environment, University of Graz, Universitaetsplatz 1, 8010 Graz, Austria

**Keywords:** HPLC/ICPQQQMS, Sulfur speciation, Taurine, Urine

## Abstract

The advent of the triple quadrupole technology to the inductively coupled plasma mass spectrometry (ICPMS) technique has allowed a strong improvement in the accuracy and detection limits of ICPMS for non-metal elements such as sulfur by removing major polyatomic interferences. Up to now, there has been no report utilizing this development for sulfur speciation in complex human biological matrices. In the present report, we show the success of HPLC-ICPQQQMS for the simultaneous determination of two major sulfur metabolites, taurine and sulfate, in human urine and serum, by direct injection without the need for sample clean-up. The optimized chromatographic method was validated, tested for robustness, and applied for investigating the intra-individual variability in taurine urinary excretion in eight healthy volunteers over a period of 8 weeks. The limit of detection and limit of quantification for taurine determination was found to be 0.2 and 0.7 pmol, respectively. The concentrations found in the analyzed group of urine samples (*n* = 64) had a range, mean, and SD of 0.6–99, 20.4, and 23.2 μg mL^−1^ for taurine, and 115–1373, 616, and 259 μg mL^−1^ for sulfate. Taurine was found to exhibit a much higher intra-individual variability than sulfate. The developed method can be applied in large-scale epidemiological studies and clinical studies in order to establish the potential cardioprotective effects of taurine.

**Electronic supplementary material:**

The online version of this article (10.1007/s00216-018-1251-z) contains supplementary material, which is available to authorized users.

**Graphical abstract:**
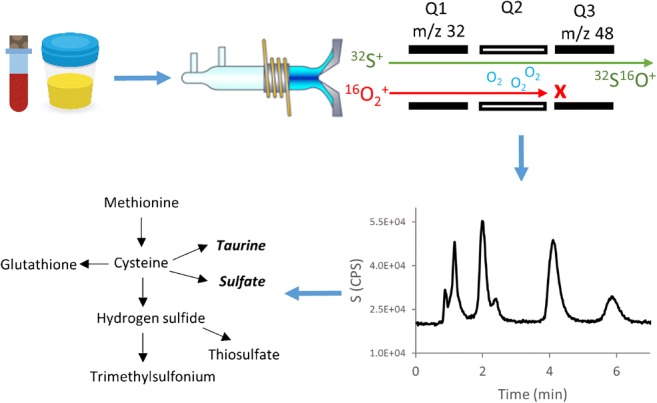
ᅟ

## Introduction

Taurine and sulfate are two major metabolites of sulfur metabolism, originating in the human body from endogenous production by the amino acid oxidation pathways [1], and also from exogenous dietary intake. Taurine is recognized as a significant compound for a variety of physiological functions [2, 3]. It was first reported to possess protective effects against hypertension [4] and stroke [5] through mouse models. These experimental findings were later supported by human epidemiological studies [6–8]. In particular, the WHO-coordinated CARDIAC (Cardiovascular Diseases and Alimentary Comparisons) study involved multiple worldwide populations and revealed an inverse correlation between the 24-h urinary excretion of taurine and ischemic heart disease markers such as total blood cholesterol, blood pressure, body mass index, and the atherogenic index [9]. Although there has been a large body of experimental evidence supporting the cardioprotective effects of taurine, the possible use of taurine as a dietary supplement or therapeutic drug in humans needs to be justified by large-scale phase 3 clinical trials which are currently missing. Such studies would benefit from fast and simple analytical methods for taurine determination in biological samples. Due to the absence of a chromophore in the chemical structure of taurine, the majority of available analytical methods for taurine analysis employ a sample pretreatment step involving a derivatization agent followed by UV or fluorescence detection [10–12]. Taurine has also been determined using an electrospray triple quadrupole mass spectrometry detector but with the use of an isotopically labeled internal standard in order to account for the matrix suppression with the electrospray ionization in biological samples [13, 14]. The use of an inductively coupled plasma mass spectrometer (ICPMS) as an HPLC detector has clear analytical advantages over the currently used detection methods, including the ability to directly analyze untreated biological samples without the requirement of a purification or derivatization step or the availability of an isotopically labeled internal standard. This simplification and expedition of the analysis is especially important in large-scale clinical trials where several thousands of samples may be involved. Up to date, there has been no report about the utilization of HPLC-ICPMS for sulfur speciation in complex human biological matrices such as urine or serum. This is mainly due to polyatomic interferences [15], especially by ^16^O^16^O^+^, which results in a background equivalent concentration of up to 9.5 mg L^−1^, and severely compromise the limit of detection [16]. In 2012, the triple quadrupole technology was introduced to ICPMS and enabled the trace analysis of many non-metal elements with major polyatomic interferences, providing an improvement in the detection limit by several orders of magnitude [16–18]. This development enables not only the utilization of the analytical advantages of ICPMS as a quantitative tool for the analysis of these elements [19] but also the exploitation of its potential as a tool for element-selective biomarker discovery and metabolic profiling [20, 21]. In the present work, we show the first success of HPLC-ICPQQQMS for sulfur speciation in complex human matrices such as urine and serum. The developed method for taurine and sulfate determination was used to investigate the week-to-week variability in their urinary excretion in healthy volunteers.

## Experimental

### Chemicals and reagents

Taurine and ammonium sulfate standards were obtained from Sigma-Aldrich (Steinheim, Germany). Purified water (Milli-Q water, 18.2 MΩ cm), obtained from a Milli-Q water purification system (Millipore GmbH, Vienna, Austria), was used for the preparation of standards and HPLC mobile phase. The HPLC eluents were prepared from ammonium acetate (Sigma-Aldrich, Steinheim, Germany) and the pH adjustment was performed using ammonia 25% (Carl Roth, Karlsruhe, Germany).

### Instrumentation

An Agilent 1100 HPLC system (Agilent Technologies, Waldbronn, Germany) was coupled to an inductively coupled plasma triple quadrupole mass spectrometric detector (Agilent 8800, Agilent Technologies, Waldbronn, Germany) with a capillary (PEEK, 0.127-mm ID, 21 cm) connecting the chromatographic column with the nebulizer on the spray chamber of the ICPQQQMS. The HPLC system was equipped with an autosampler ALS G1367C, a degasser G1379A, a quaternary pump G1311A, a sample chiller ALSTherm G1330B, and a column compartment COLCOM G1316A. The ICPQQQMS system was equipped with an AriMist PEEK® nebulizer, a Scott-type spray chamber, Ni/Cu sampler and skimmer cones, and a 2.5-mm quartz plasma torch. Oxygen as a reaction gas at a flow rate of 0.3 mL min^−1^ was used to produce the mass shift 32–> 48 and 34–> 50 for monitoring the ^32^S and ^34^S isotopes, respectively. A 1.0 μg L^−1^ multi-element tuning solution was used for tuning the ICPQQQMS. This was performed in the MS/MS mode by monitoring the mass transition 89–> 105 (Y–> YO), 59–> 59 (Co), and 205–> 205 (Tl) (see Electronic Supplementary Material (ESM) Fig. S1). The oxide ratios and doubly charged ion formation were monitored through measuring CeO/Ce and Ce^2+^/Ce^+^, and were < 1.5 and < 2.0%, respectively.

### Chromatographic conditions

A Hamilton anion-exchange PRP-X100 column (150 mm × 2.1 mm, 5 μm) was used for the separation of the sulfur compounds, at a temperature of 50 °C and a mobile-phase flow rate of 0.5 mL min^−1^. A step gradient consisting of A= Milli-Q water and B = 100 mM ammonium acetate buffer with pH = 9.0 (adjusted with ammonia) was used as follows: 0–5 min 7% B; 5–10 min 100% B; 10–15 min 7% B. The injection volume for urine and serum was 0.3 and 0.5 μL, respectively.

### Volunteer recruitment and sample collection

Urine samples were collected from eight healthy volunteers as previously described [22]. Briefly, the study group consisted of three females and five males with an age range, mean, and SD of 18–60, 37, and 13 years, respectively. The volunteers were living in the city of Graz, Austria. All volunteers were non-smokers except for volunteers G and H. The volunteers gave informed consent and all procedures were in accordance with the Declaration of Helsinki. Ethical approval for the present study was obtained from the ethical committee at the University of Graz (GZ. 39/46/63). Each volunteer collected one morning urine sample (first-pass of the day) on Corning® polypropylene 300-mL sample collection containers (Corning, NY, USA). Portions of the samples (ca. 4.5 mL each) were transferred to 5-mL Eppendorf® tubes (Eppendorf, Vienna, Austria) and stored at − 80 °C until the time of analysis. The volunteers were asked to record dietary information throughout the sample collection phase, by filling a general food consumption table involving a typical Austrian diet, adapted from Pestitschek et al. [23]. For method validation in serum, we used eight serum samples collected from eight healthy non-smoking volunteers (age range 51–60, mean (SD) 54 [5]). Blood was collected in BD Vacutainer™ serum collection tubes, allowed to clot at room temperature for 30–60 min, and serum was separated by centrifugation at 5000 rpm for 15 min. A volume of 1.0 mL serum was transferred into 1.5-mL Eppendorf® tubes, freeze-dried, and stored at 4 °C until the time of analysis.

### Sample preparation

Urine was thawed at 4 °C, vortex-mixed, then withdrawn with 2-mL polypropylene syringes (NORM-JECT®, Henke Sass Wolf, Tuttlingen, Germany) and filtered using 0.2-μm Nylon syringe filters (Chromafil® Xtra PA-20/13, Macherey-Nagel GmbH, Dueren, Germany) into 0.7-mL polypropylene HPLC vials (Bruckner, Linz, Austria) with polyethylene snap-caps (Bruckner, Linz, Austria) before injection into the HPLC-ICPQQQMS system. Freeze-dried serum was reconstituted in 1.0 mL Milli-Q water, transferred to 2.0-mL Eppendorf® tubes, and mixed with 1.0 mL HPLC grade acetonitrile (VWR® international, Leuven, Belgium) in order to precipitate the proteins. The mixture was vortex-mixed and centrifuged at 21,380×*g* and 4 °C for 20 min with a SciLogex D3024R microcentrifuge (SciLogex®, Rocky Hill, NY, USA). The supernatant was transferred to 0.7-mL polypropylene HPLC vials with polyethylene snap-caps and injected into the HPLC-ICPQQQMS system.

## Results and discussion

### Chromatographic separation

The chromatographic conditions were thoroughly optimized in order to ensure a complete and robust separation of taurine from the variable and complex urine matrix. Several eluents were tested, including ammonium phosphate, ammonium citrate, and ammonium bicarbonate. Due to the positively charged amino group on taurine (pKa = 9.1), it was necessary to work under basic pH conditions in order to allow a net negative charge on taurine and a sufficient retention on the anion-exchange column. Furthermore, we observed that singly charged eluents allowed a milder elution strength for taurine and therefore the best retention and separation from the matrix. Therefore, the best results were achieved with ammonium acetate at pH = 8.5–9.5. Subsequently, systematic testing of this eluent was performed under these conditions (ESM Fig. S2). The optimal conditions providing separation from the sample matrix while maintaining an acceptable retention factor were found to be 7 mM ammonium acetate at pH = 9.0. A step gradient with up to 100 mM ammonium acetate was included in order to elute the high amounts of sulfate in urine.

### ICPQQQMS detection

**Fig. 1:**
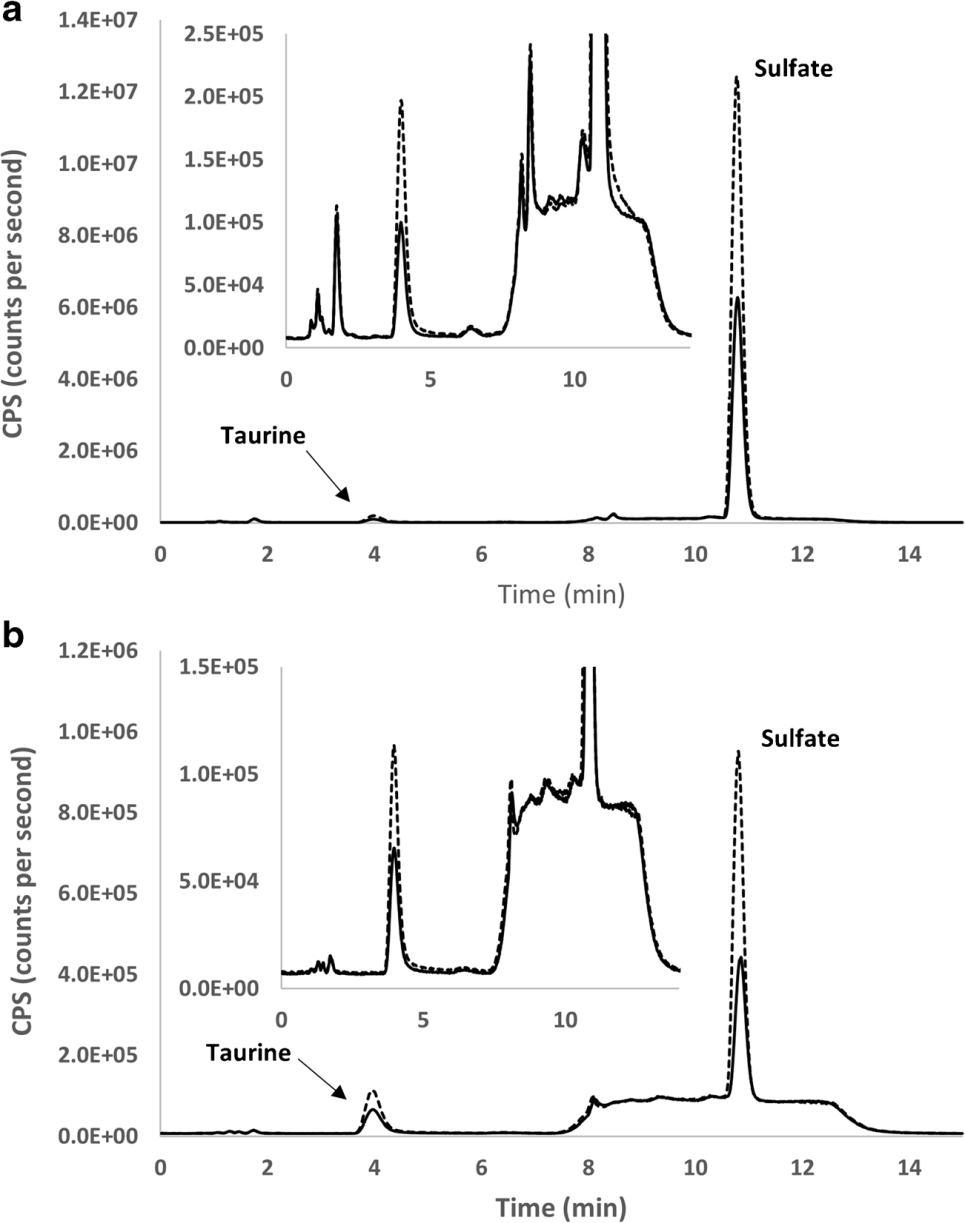
Taurine und sulfate in human urine (**a**) and serum (**b**). 0.1 μL of urine was spiked with 0.1 μL of a mixture containing 12.5 μg S mL^-1^ taurine and 500 μg S mL^-1^ sulfate. 0.3 μL of serum was spiked with 0.3 μL of a mixture containing 2.0 μg S mL^-1^ taurine and 15 μg S mL^-1^ sulfate. For chromatographic conditions, see text

A unique advantage of the ICPMS detector is that it provides an element-focused approach and is therefore particularly an attractive analytical tool in element-based metabolomics. The optimized chromatographic conditions can be used for the determination of sulfate and taurine in urine as well as in serum matrix (Fig. 1). The developed method involves minimal sample preparation, including simple filtration for urine and protein precipitation for serum. The elimination of the need for derivatization or sample purification enables a higher throughput of analysis, which is especially suitable for large-scale epidemiological studies. Furthermore, in contrast with the electrospray ionization triple quadrupole mass spectrometric detector (ESIQQQMS), the ICPMS detector is known to be much less prone to matrix effects. This is shown by the agreement in quantification results between the external standardization and the standard addition method (Table 1). According to a previous report, it was possible to achieve a low detection limit for total sulfur determination in a water matrix (0.2 ng S mL^−1^), using ICPMS equipped with a dynamic reaction cell with a single quadrupole mass analyzer [24]. This was implemented by using oxygen as the reaction gas and monitoring *m*/*z* = 48, which is associated with a much lower background compared to *m*/*z* = 32 [24]. This approach, however, is not suitable for biological matrices due to the interferences by ^31^P^16^O^1^H^+^, ^36^Ar^12^C^+^, and ^48^Ca^+^. The use of a triple quadrupole mass analyzer eliminates these interferences while maintaining an equally low detection limit [16].

**Table 1:** Validation of taurine and sulfate determination in human urine and serum. Concentrations are expressed as μg S ml^-1^

Urine^1)^	**Taurine**						**Sulfate**					
Sample	External standardization			Standard addition^2)^	Recovery (%)^3)^		External standardization			Standard addition	Recovery (%)	
	Mean	Intra-day precision^4)^	Inter-day precision		L1	L2	Mean	Intra-day precision	Inter-day precision		L1	L2
1	10	2.1	3.7	10	107 ± 10	99 ± 2	262	2.1	3.2	278	107 ± 5	105 ± 1
2	20	2.2	5.0	21	112 ± 14	102 ± 4	633	2.2	4.3	691	111 ± 7	107 ± 3
3	17	2.7	4.6	16	99 ± 10	103 ± 2	559	2.7	3.4	550	102 ± 6	106 ± 3
4	24	2.4	6.2	24	98 ± 7	101 ± 1	638	1.6	4.6	655	106 ± 4	107 ± 1
5	10	3.4	1.7	10	105 ± 5	101 ± 1	734	1.5	1.8	770	111 ± 4	108 ± 2
6	8.9	1.1	4.5	9.3	95 ± 3	100 ± 2	773	1.0	3.6	840	100 ± 2	106 ± 3
7	13	1.0	4.2	13			987	1.0	3.2	1008		
8	11	1.7	4.6	12			1085	1.7	4.0	1259		
Serum	**Taurine**						**Sulfate**					
Sample	External standardization			Standard addition^5)^	Recovery (%)^6)^		External standardization			Standard addition	Recovery (%)	
	Mean	Intra-day precision	Inter-day precision		L1	L2	Mean	Intra-day precision	Inter-day precision		L1	L2
1	2.2	0.7	8.0	2.0	95 ± 11	103 ± 1	4.6	1.6	10.3	4.6	104 ± 1	104 ± 1
2	3.2	2.9	3.2	3.0	112 ± 7	100 ± 6	6.3	1.4	8.8	6.3	109 ± 2	101 ± 1
3	3.6	2.8	2.6	3.5	96 ± 7	101 ± 7	7.9	1.2	5.6	7.9	107 ± 2	104 ± 1
4	1.5	1.9	10.3	1.3	92 ± 13	98 ± 3	6.4	1.4	7.0	6.4	103 ± 1	102 ± 1
5	2.0	1.3	9.9	2.2	88 ± 11	101 ± 1	5.4	1.0	7.0	5.4	104 ± 1	102 ± 1
6	2.4	2.9	5.4	2.5	109 ± 4	96 ± 2	6.6	1.3	5.5	6.6	109 ± 2	100 ± 1
7	1.7	1.4	6.9	1.6			5.8	0.9	3.7	5.8		
8	2.3	1.9	6.2	2.3			5.1	1.9	3.5	5.1		

1) The specific gravities of urine samples 1-8 are 1.012, 1.015, 1.016, 1.019, 1.021, 1.024, 1.027, and 1.031, respectively. The concentrations reported are the measured raw concentrations

2) The standard addition was performed for urine by co-injecting standard solutions of taurine and sulfate with concentrations within the range of 2.5-15, and 125-750 μg S ml^-1^, respectively

3) The recovery testing was performed by manually spiking 900 μL of urine with 100 μL of standard solutions, producing a spiking level of 5 μg S ml^-1^ (L1) and 25 μg S ml^-1^ (L2) of taurine, and 250 μg S ml^-1^ (L1) and 1250 μg S ml^-1^ (L2) of sulfate

4) Precision is expressed as the %RSD

5) The standard addition was performed for serum by co-injecting standard solutions of taurine and sulfate with concentrations within the range of 0.5-5.0, and 5.0-20 μg S ml^-1^, respectively

6) The recovery testing was performed by manually spiking 300 μL of serum following protein precipitation (see text) with 75 μL of standard solutions, producing a spiking level of 1.0 μg S ml^-1^ (L1) and 4.0 μg S ml^-1^ (L2) of taurine, and 10 μg S ml^-1^ (L1) and 40 μg S ml^-1^ (L2) of sulfate

### Method validation

**Fig. 2:**
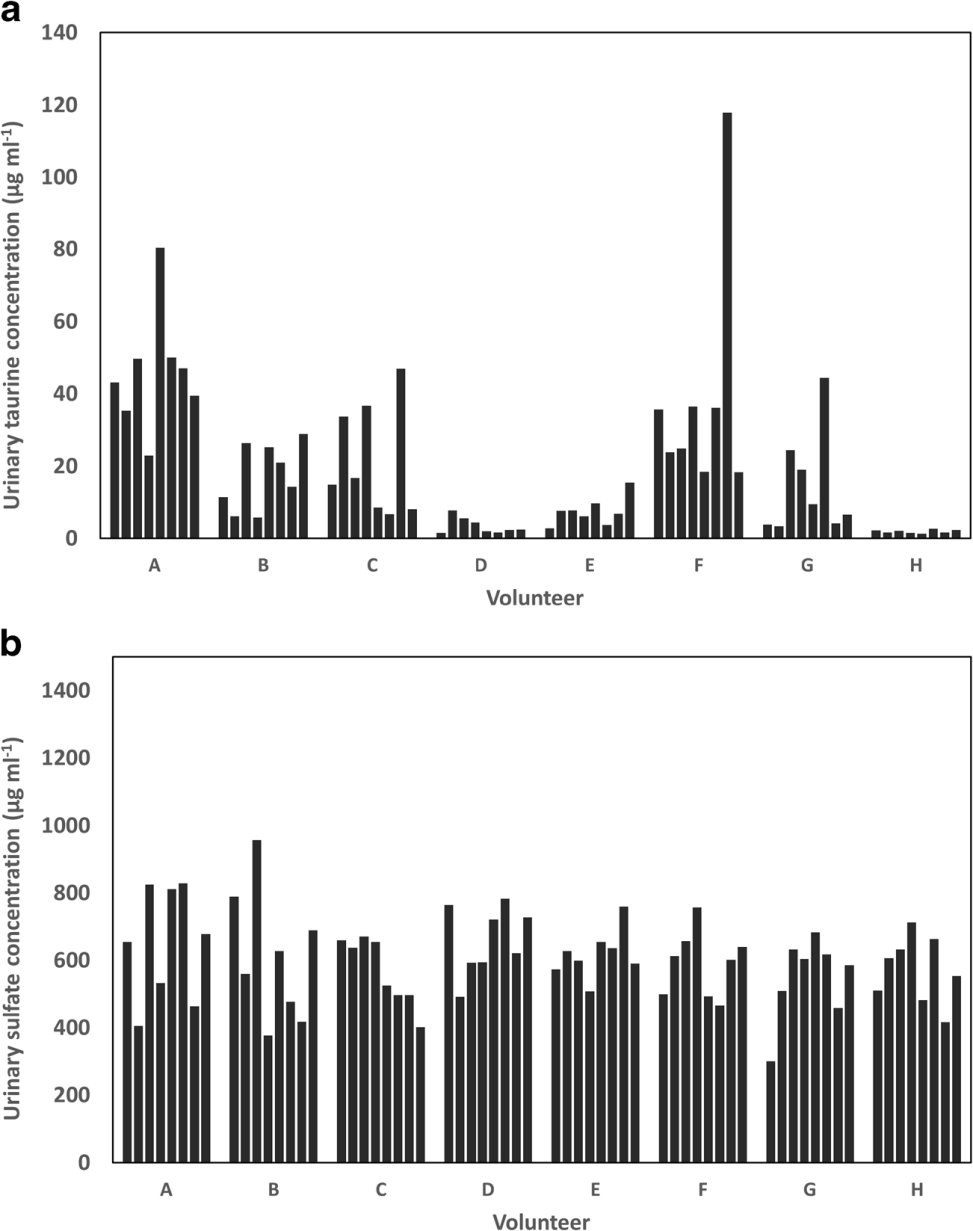
The inter-week variability in the urinary excretion of taurine (**a**) and sulfate (**b**) in eight healthy volunteers. All concentrations were reported in μg S mL^−1^ and adjusted to specific gravity. For details about the volunteers, see text

The developed chromatographic method has a limit of detection of 0.2 pmol (calculated based on the method of the standard error of the *y*-intercept of a calibration curve recorded in the range of 0.5–4.8 pmol (0.5–5.0 μg S mL^−1^), and an injection volume of 0.3 μL). The repeatability of the peak area of a taurine standard in water prepared at a concentration of 0.1 μg S mL^−1^ (0.9 pmol) was found to be 7.6% (*n* = 6) (ESM Fig. S3). Due to the absence of a certified urine or serum for taurine and sulfate concentration, accuracy was assessed by spiking a series of eight urine and serum samples with two different levels of taurine and sulfate and by determining the recovery of the spiking (Table 1). Moreover, the urinary and serum levels of taurine and sulfate in the analyzed samples (Table 1 and Fig. 2) were in accordance with the values previously reported for healthy control groups [14, 25–29]. For example, Gamagedara et al. analyzed urine samples of 12 healthy subjects by HPLC-ESIQQQMS and the concentration of taurine had a range, mean, and SD, respectively, of 0.047–8.9, 1.8, and 2.35 mM (1.5–258, 58.6, and 75.2 μg mL^−1^) [14], while Tcherkas et al. found a mean ± SEM (standard error of the mean) taurine concentration in serum samples from 16 healthy volunteers 102.5 ± 17.3 nmol mL^−1^ (3.3 ± 0.55 μg mL^−1^, analyzed by means of HPLC with an electrochemical detection [28]. The average concentrations of sulfate in healthy human subjects were reported to be 24 mM (768 μg mL^−1^) for urine [26] and 0.3 mM (9.6 μg mL^−1^) for serum [29], respectively. We assessed the intra-day and inter-day repeatability of our measurements (Table 1). The time interval between the analyses of the three inter-day precision replicates was about 1 week. During this period, we kept the urine and serum samples stored in the fridge (4–8 °C), in order to give us an indication of taurine stability under these conditions. No observable loss of taurine was found, indicating that taurine is stable for at least 2 weeks when stored at (4–8 °C) (see inter-day precision data in Table 1). Due to the complexity and variability of the sulfur matrix in human urine, it was crucial to ensure that the developed method is robust enough to be applied under various settings. We tested the robustness of the developed method by requantifying five morning urine samples with different specific gravities from five different volunteers while varying chromatographic conditions such as mobile-phase pH, column temperature, buffer concentration, and injection volume. The variation in the calculated concentration was generally within a ± 10% margin (ESM Fig. S4). Furthermore, the chromatographic separation was shown to be robust in all of the 64 urine samples analyzed from the eight different volunteers in the present study (for examples, see ESM Fig. S5). The present method allows simultaneous determination of taurine and sulfate. In case only taurine is of interest, it is possible to add barium chloride to a final concentration of 50 mM, which was found to remove > 99% of sulfate in urine (barium sulfate solubility in water at 20 °C is 0.3 μg S mL^−1^). This allows shortening the chromatographic runtime to 5 min, and increasing sample throughput to ca.12 samples/h. This added sulfate precipitation step can be conveniently incorporated in the urine filtration step prior to HPLC injection.

### The intra-individual variability in the urinary excretion of taurine and sulfate

Although serum is widely used in epidemiological studies as an indicator of taurine status in the human body [6], urine is less expensive and more convenient to collect. However, there is currently no report that investigates the long-term stability of taurine levels in human urine and the intra-individual variability for its excretion. This investigation is important before making interpretations of taurine concentration in urine as an indicator for taurine body status. As a preliminary study, we determined sulfate and taurine concentration in 64 urine samples collected from eight volunteers over 8 weeks. In order to account for variation in fluid intake, the concentrations were normalized according to specific gravity based on the equation by Levine et al. [30]:

$$ {C}_{\mathrm{norm}}=\frac{\mathrm{spec}.{\mathrm{grav}}_{\mathrm{mean}}-1}{\mathrm{spec}.{\mathrm{grav}}_{\mathrm{sample}}-1}\times {C}_{\mathrm{sample}} $$

Our results show a higher intra-individual and inter-individual variability in taurine urinary excretion relative to the excretion of sulfate, the other oxidation product of cysteine (Fig. 2). This difference in the stability of urinary excretion between sulfate and taurine can be explained by a low contribution of the endogenously produced taurine to total urinary taurine and therefore a higher dependency of the total urinary taurine on variable dietary taurine intake. Indeed, it was shown that the enzymatic activity of the enzyme responsible for the endogenous production of taurine (cysteine sulfinic acid decarboxylase; EC 4.1.1.29) is the rate-limiting factor for taurine biosynthesis [31]. Furthermore, we observed that volunteers A and F who had higher taurine excretion had relatively higher intake of fish, which is known to be a rich source of taurine [32]. In conclusion, the ICPQQQMS detection was shown to be applicable to complex human biological matrices for sulfur speciation. The developed chromatographic method for taurine analysis in human urine and serum offers the advantages of speed and simplicity, and can be used in future large-scale clinical trials in order to establish the cardioprotective roles of taurine in humans and justify its potential usage as a dietary supplement. Further applications of the HPLC-ICPQQQMS for sulfur speciation in human biological matrices will certainly follow.

## Electronic supplementary material

ESM 1 (PDF 858 kb)
