# Supplementary material for: Sulfur speciation by HPLC-ICPQQQMS in complex human biological samples: taurine and sulfate in human serum and urine
Source: Anal Bioanal Chem. 2018 Jul 30;410(26):6787–93. doi: 10.1007/s00216-018-1251-z (PMC6132542; doi:10.1007/s00216-018-1251-z)
Supplement: Supplementary file 1 — (PDF 858 kb) [file 216_2018_1251_MOESM1_ESM.pdf]

Analytical and Bioanalytical Chemistry

Electronic Supplementary Material

Sulfur speciation by HPLC-ICPQQQMS in complex human biological samples: taurine and sulfate in human serum and urine

Bassam Lajin, Walter Goessler

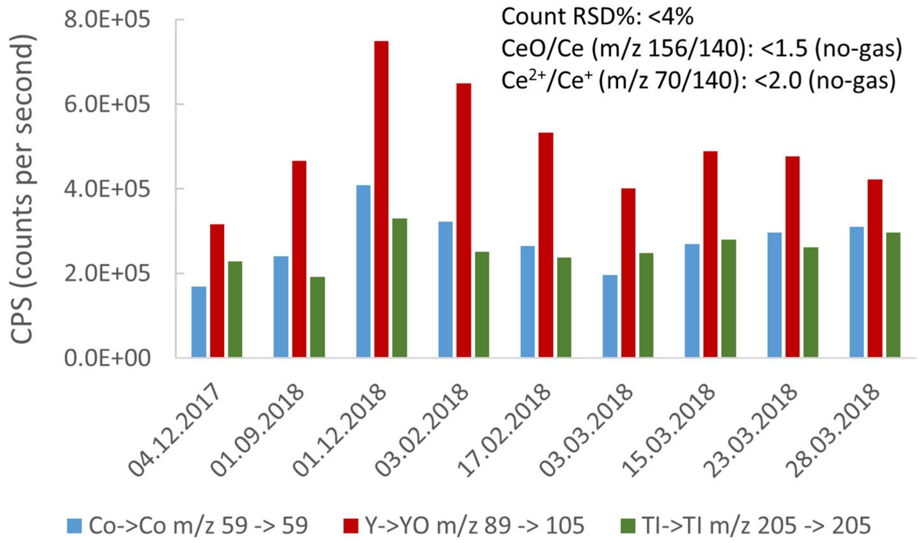

Fig. S1 Performance control over the period of method development. A multi-element tuning solution containing 1.0 ng mL^{-1} of the designated elements is used to check the performance of the ICPQQQMS instrument in the total element analysis mode. The graph shows the counts per second of the designated elements measured using oxygen as the reaction cell gas. For instrumental conditions see text

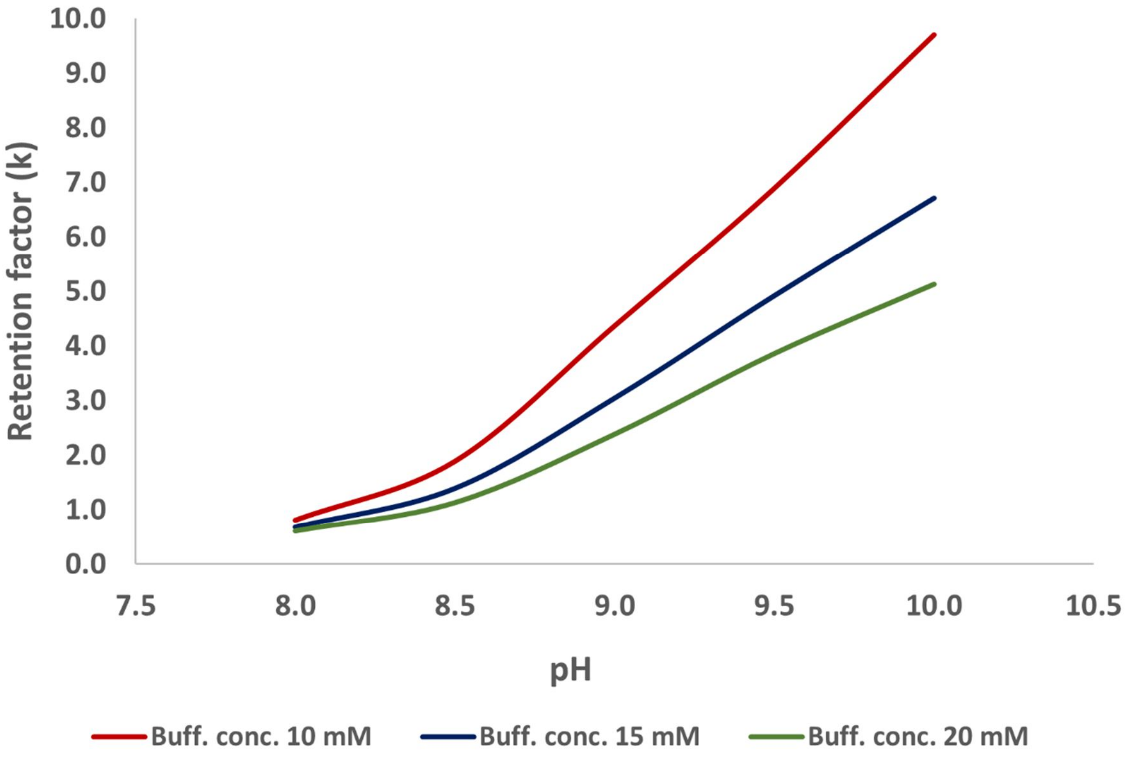

Fig. S2 Investigating the retention behavior of taurine with varying mobile phase pH and buffer concentration (ammonium acetate). For chromatographic conditions, see text

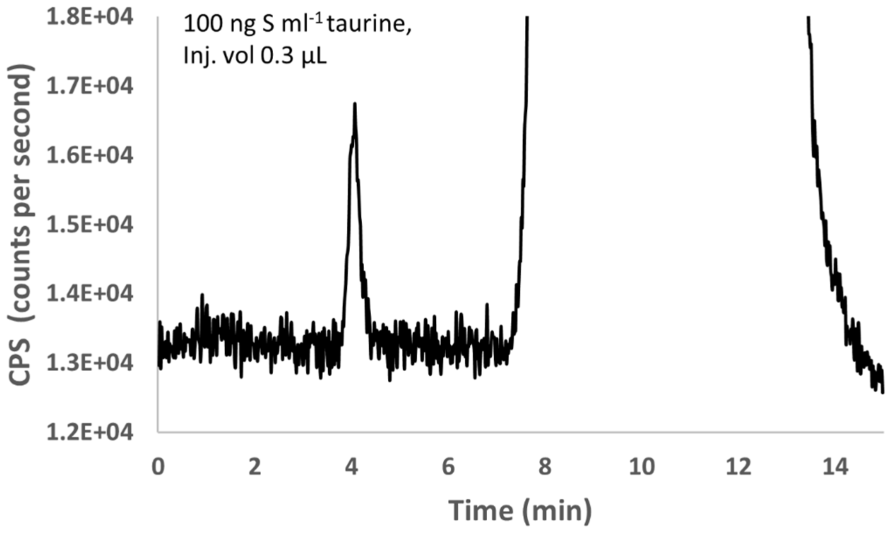

Fig. S3 A chromatogram of a taurine standard in water containing 100 ng S mL^{-1}. The injection volume is 0.3 µL. The peak area RSD% (n=6) was 7.6%. The calculated limit of quantification is 60 ng S mL^{-1} (based on the method of the standard error of the y-intercept, see text for details)

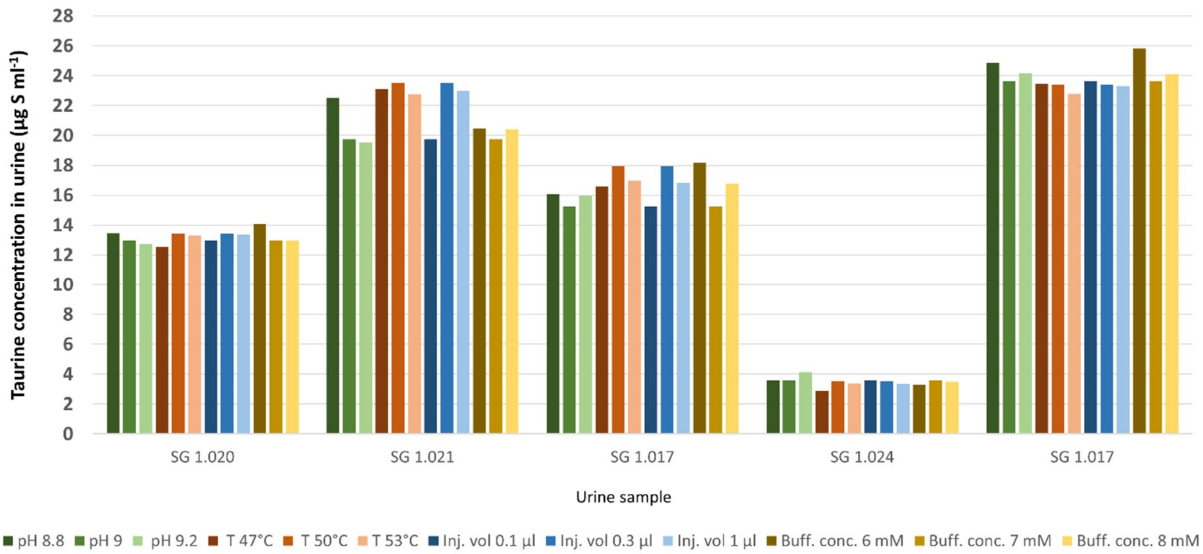

Fig. S4 Robustness testing. Taurine was re-quantified in five morning urine samples from five different volunteers while varying one of 4 variables at a time (buffer pH (8.8-9.2), column temperature (47-53 °C), injection volume (0.1-1 µL), and buffer concentration (6-8 mM)). The specific gravities (SG) of the urine samples are included on the graph as indication of the matrix concentration. For chromatographic conditions, see text

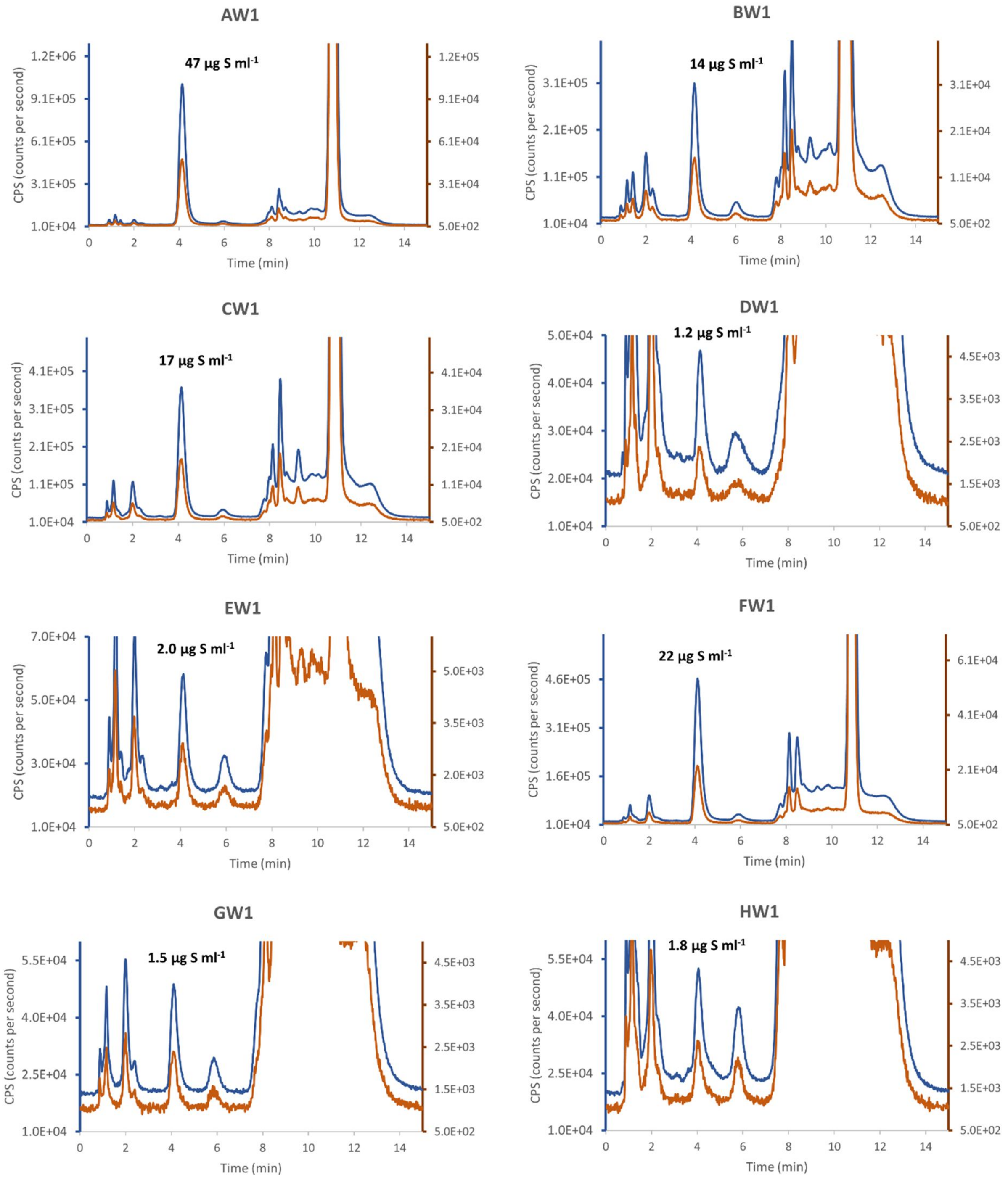

Fig. S5 Example chromatograms showing the urine samples calculated at week 1 (W1) calculated from 8 healthy volunteers (A-H) for the investigation of inter-week variability in taurine and sulfate excretion Blue: ^{32}S trace, orange: ^{34}S
